# Non-motor symptom burden in patients with Parkinson’s disease with impulse control disorders and compulsive behaviours: results from the COPPADIS cohort

**DOI:** 10.1038/s41598-020-73756-z

**Published:** 2020-10-09

**Authors:** S. Jesús, M. A. Labrador-Espinosa, A. D. Adarmes, C. Méndel-Del Barrio, J. C. Martínez-Castrillo, A. Alonso-Cánovas, P. Sánchez Alonso, S. Novo-Ponte, M. G. Alonso-Losada, N. López Ariztegui, J. C. Segundo Rodríguez, M. I. Morales, I. Gastón, F. Lacruz Bescos, P. Clavero Ibarra, J. Kulisevsky, J. Pagonabarraga, B. Pascual-Sedano, P. Martínez-Martín, D. Santos-García, P. Mir, COPPADIS Study Group, M. Aguilar, M. Almeria, M. Álvarez Sauco, S. Arnáiz, S. Arribas, A. Ascunce Vidondo, N. Bernardo, H. Bejr-Kasem, M. Blázquez Estrada, M. Botí, C. Borrue, C. Cabello González, A. Cámara Lorenzo, F. Carrillo, F. Carrillo Padilla, E. Casas, M. J. Catalán, A. Cortina Fernández, A. Cots Foraster, A. Crespo Cuevas, E. Cubo, M. Díez-Fairen, S. Escalante, E. Erro, O. de Fábregues-Boixar, N. Fernández Guillán, P. Gámez, M. Gallego, J. García Caldentey, C. García Campos, J. M. García Moreno, M. P. Gómez Garre, J. González Aloy, J. González Ardura, B. González García, M. J. González Palmás, G. R. González Toledo, A. Golpe Díaz, M. Grau Solá, G. Guardia, A. Horta-Barba, J. Infante, M. Kurtis, J. Hernández Vara, C. Labandeira, M. Lage Castro, I. Legarda, L. M. López Díaz, L. López Manzanares, B. López Seoane, Y. Macías, M. Mata, G. Martí Andres, M. J. Martí, D. McAfee, M. T. Meitín, M. Menéndez González, J. Miranda Santiago, A. Moreno Diéguez, V. Nogueira, A. Novo Amado, C. Ordás, P. Pastor, J. M. Paz González, I. Pareés, A. Pérez Fuertes, R. Pérez Noguera, L. Planellas, M. A. Prats, C. Prieto Jurczynska, M. Pueyo, V. Puente, N. Redondo Rafales, L. Rodríguez Méndez, A. B. Rodríguez Pérez, F. Roldán, M. Ruíz De Arcos, J. Ruíz Martínez, M. Sánchez-Carpintero, G. Sánchez Díez, A. Sánchez Rodríguez, P. Santacruz, M. Seijo, A. Serarols, M. Sierra Peña, B. Solano, E. Suárez-Castro, J. P. Tartari, C. Valero, L. Vargas, R. Vázquez Gómez, L. Vela, C. Villanueva, B. Vives, M. D. Villar

**Affiliations:** 1 Unidad de Trastornos del Movimiento, Servicio de Neurología Y Neurofisiología Clínica, Instituto de Biomedicina de Sevilla, Hospital Universitario Virgen del Rocío/CSIC/Universidad de Sevilla, Av. Manuel Siurot s/n. 41013, Seville, Spain; 2 Centro de Investigación Biomédica en Red Sobre Enfermedades Neurodegenerativas (CIBERNED), Barcelona, Spain; 3 Hospital Universitario Ramón Y Cajal, Madrid, Spain; 4 Hospital Universitario Puerta del Hierro, Madrid, Spain; 5 Hospital Meixoeiro, Complejo Hospitalario Universitario de Vigo, Vigo, Spain; 6 Complejo Hospitalario de Toledo, Toledo, Spain; 7 Complejo Hospitalario de Navarra, Pamplona, Navarra Spain; 8 Unidad de Trastornos del Movimiento, Servicio de Neurología, Hospital de La Santa Creu I Sant Pau, Barcelona, Spain; 9 Centro Nacional de Epidemiología, Instituto de Salud Carlos III, Madrid, Spain; 10 Complejo Hospitalario Universitario de A Coruña (CHUAC), A Coruña, Spain; 11 Faculty of Health Sciences, Universitat Oberta de Catalunya (UOC), Barcelona, Spain; 12 Hospital Universitari Mutua de Terrassa, Terrassa, Barcelona, Spain; 13 Hospital General Universitario de Elche, Elche, Spain; 14 Complejo Asistencial Universitario de Burgos, Burgos, Spain; 15 Hospital de Tortosa Verge de la Cinta (HTVC), Tortosa, Tarragona Spain; 16 Hospital Universitario Central de Asturias, Oviedo, Spain; 17 Hospital Infanta Sofía, Madrid, Spain; 18 Hospital Clınic de Barcelona, Barcelona, Spain; 19 Hospital Universitario de Canarias, San Cristóbal de la Laguna, Santa Cruz de Tenerife, Spain; 20 Hospital Clínico San Carlos, Madrid, Spain; 21 Complejo Hospitalario Universitario de Ferrol (CHUF), Ferrol, A Coruña, Spain; 22 Institut d’Assistencia Sanitaria (IAS) – Institut Catala de la Salud, Girona, Spain; 23 Hospital del Mar, Barcelona, Spain; 24 Hospital Universitario Vall d’Hebron, Barcelona, Spain; 25 Hospital La Princesa, Madrid, Spain; 26 Centro Neurologico Oms 42, Palma de Mallorca, Spain; 27 Hospital Universitario Virgen Macarena, Sevilla, Spain; 28 Hospital Universitario Lucus Augusti (HULA), Lugo, Spain; 29 Complejo Hospitalario Universitario de Pontevedra (CHOP), Pontevedra, Spain; 30 Consorci Sanitari Integral, Hospital Moises Broggi, Sant Joan Despı, Barcelona, Spain; 31 Hospital Universitario Marqués de Valdecilla, Santander, Spain; 32 Hospital Ruber Internacional, Madrid, Spain; 33 Hospital Universitario Son Espases, Palma de Mallorca, Spain; 34 Hospital Da Costa de Burela, Lugo, Spain; 35 Fundacion Hospital de Alcorcon, Madrid, Spain; 36 University of Pennsylvania, Philadelphia, PA USA; 37 Hospital Rey Juan Carlos, Madrid, Spain; 38 Hospital Universitario Donostia, San Sebastián, Spain; 39 Hospital Arnau de Vilanova, Valencia, Spain

**Keywords:** Neurological disorders, Neurology

## Abstract

The study was aimed at analysing the frequency of impulse control disorders (ICDs) and compulsive behaviours (CBs) in patients with Parkinson’s disease (PD) and in control subjects (CS) as well as the relationship between ICDs/CBs and motor, nonmotor features and dopaminergic treatment in PD patients. Data came from COPPADIS-2015, an observational, descriptive, nationwide (Spain) study. We used the validated Questionnaire for Impulsive-Compulsive Disorders in Parkinson's Disease-Rating Scale (QUIP-RS) for ICD/CB screening. The association between demographic data and ICDs/CBs was analyzed in both groups. In PD, this relationship was evaluated using clinical features and treatment-related data. As result, 613 PD patients (mean age 62.47 ± 9.09 years, 59.87% men) and 179 CS (mean age 60.84 ± 8.33 years, 47.48% men) were included. ICDs and CBs were more frequent in PD (ICDs 12.7% vs. 1.6%, *p* < 0.001; CBs 7.18% vs. 1.67%, *p* = 0.01). PD patients had more frequent previous ICDs history, premorbid impulsive personality and antidepressant treatment (*p* < 0.05) compared with CS. In PD, patients with ICDs/CBs presented younger age at disease onset, more frequent history of previous ICDs and premorbid personality (*p* < 0.05), as well as higher comorbidity with nonmotor symptoms, including depression and poor quality of life. Treatment with dopamine agonists increased the risk of ICDs/CBs, being dose dependent (*p* < 0.05). As conclusions, ICDs and CBs were more frequent in patients with PD than in CS. More nonmotor symptoms were present in patients with PD who had ICDs/CBs compared with those without. Dopamine agonists have a prominent effect on ICDs/CBs, which could be influenced by dose.

## Introduction

Impulse control disorders (ICDs) and compulsive behaviours (CBs) are known conditions that frequently appear in patients with Parkinson’s disease (PD). Within the ICDs spectrum, pathological gambling, compulsive shopping, hypersexuality and compulsive eating are included. CBs are those related to the performing of aimless repetitive stereotyped complex tasks, known as punding, hobbysm, excessive and aimless wandering (“walkabout”) and dysregulation dopaminergic syndrome (DDS)^1^.

The etiopathology of these disorders is not simple. Demographic factors, premorbidities, comorbidities and genetic factors can influence the risk of ICDs and CBs^2–5^. Comorbidities such as tobacco consumption and depression are well-established factors linked to ICDs. However, the directionality of the association between ICDs and affective disorders is not still clear^6–8^. Epidemiologic factors such as sex, age and age at onset could influence the incidence of ICDs and CBs^1^. Nevertheless, it is controversial whether these disorders are exclusively linked to the first years of disease evolution when patients still have an integrum ventral striatum^2^, or whether they are associated with longer exposure to dopamine treatment^3^. On the other hand, estimated prevalence rates across countries range from 10 to 39%^4^. This wide range depends on the study characteristics, but also on the population features, being therefore important the assessment of specific populations.

In addition, it is known that dopaminergic replacement therapy (DRT) plays an important role in ICDs and CBs, given their prevalence in untreated de novo patients is the same as in control subjects (CS)^8^. Thus, under external dopaminergic stimulation by DRT, the regulation of mesocorticolimbic pathway activity might be inappropriate, inducing an aberrant response to reward tasks and impulsivity traits^9^. Despite this fact, not all patients undergoing DRT treatment have ICDs or CBs, suggesting the action of a neurobiological substrate leading to individual susceptibility^5^.

Dopamine agonists (DA) have been shown to be a principal drug involved in ICDs, and the association appears to be dose dependent^10,11^. However, there is controversy regarding DA dose and ICDs, given it is not fully understood whether the ICDs are associated with the DA dose alone or in combination with the dose of levodopa and other dopaminergic drugs^2,3,12^.

Parkinsonian features have also been associated with ICDs. Not only can cognitive performance in patients with ICDs be impaired, but executive dysfunction as well as impairment in the reward-related decision process and set shifting can be present^6,13,14^.

Taking into account the factors influencing ICDs and CBs in PD, we sought to assess clinical features linked to these disorders in the COPPADIS-2015 population of PD and CS. We especially aimed to evaluate the impact of DRT and the influence of clinical features on these disorders in the PD population.

## Methods

### Participants

The participant data was drawn from the baseline assessment of the COPPADIS-2015, an observational, descriptive, 5-year follow-up, nationwide (Spain) evaluation study^15^. The project is conducted in accordance with the standards for Good Clinical Practice and the fundamental ethical principles established in the Declaration of Helsinki. Approval of the Ethics Committee at each center was obtained (see Supplementary Material Table 6) and informed consent was obtained for each participant. COPPADIS-2015 was classified by the AEMPS (*Agencia Española del Medicamento y Productos Sanitarios*) as a Post-authorization Prospective Follow-up study with the code COH-PAK-2014-01.

A total of 694 patients with PD (mean age 62.6 ± 8.9 years, 60.3% men) and 207 CS (mean age 61 ± 8.3 years, 49.5% men) were included in the COPPADIS study^15^.

### Assessments

The Questionnaire for Impulsive-Compulsive Disorders in Parkinson’s Disease Rating Scale (QUIP-RS) was assessed in both patients with PD and CS for screening of ICDs and CBs (cutoff points: gambling ≥ 6, shopping ≥ 8, sex ≥ 8, eating ≥ 7, hobbysm-punding ≥ 7)^16^. For DDS, we used the investigator criterion, given there is no established cut-off.

Demographic data concerning educational level, marital status, smoking, alcohol intake, premorbid impulsive personality, previous ICDs and family history of ICDs were collected for both groups (patients with PD and CS).

### Data analysis

In the PD group, a cross-sectional assessment was performed for each patient according to the study methodology^15^. Baseline data were used for the analysis.

Subanalyses of the group with an age at disease onset younger than 50 years and of the DA treatment group were also performed.

Parametric univariate tests (t-test and chi-squared test) and nonparametric univariate tests (Wilcoxon test) were used to compare demographics and QUIP-RS results between the CS and the patients with PD. A logistic regression analysis (controlling for sex and age) was also used.

In the PD group analysis, univariate and multivariate analysis were used to compare demographic variables, motor and nonmotor symptoms and dopaminergic treatment variables between patients with PD with and without ICDs/CBs. While controlling for age, age at disease onset and disease progression time, a multivariate linear regression analysis was used for the quantitative variables and a logistic regression analysis was employed for the categorical variables. Finally, the association between ICDs severity and treatment was analysed using the QUIP-RS score as the outcome variable and dopaminergic treatment variables (type of treatment and levodopa-equivalent daily dose) as independent variables in multivariate linear regressions (controlling for age, age at disease onset and disease progression time). All the statistical analyses were conducted in R for Statistical Computing, version 3.5.1.

## Results

### Participants and prevalence of ICDs and CBs

**Figure 1:**
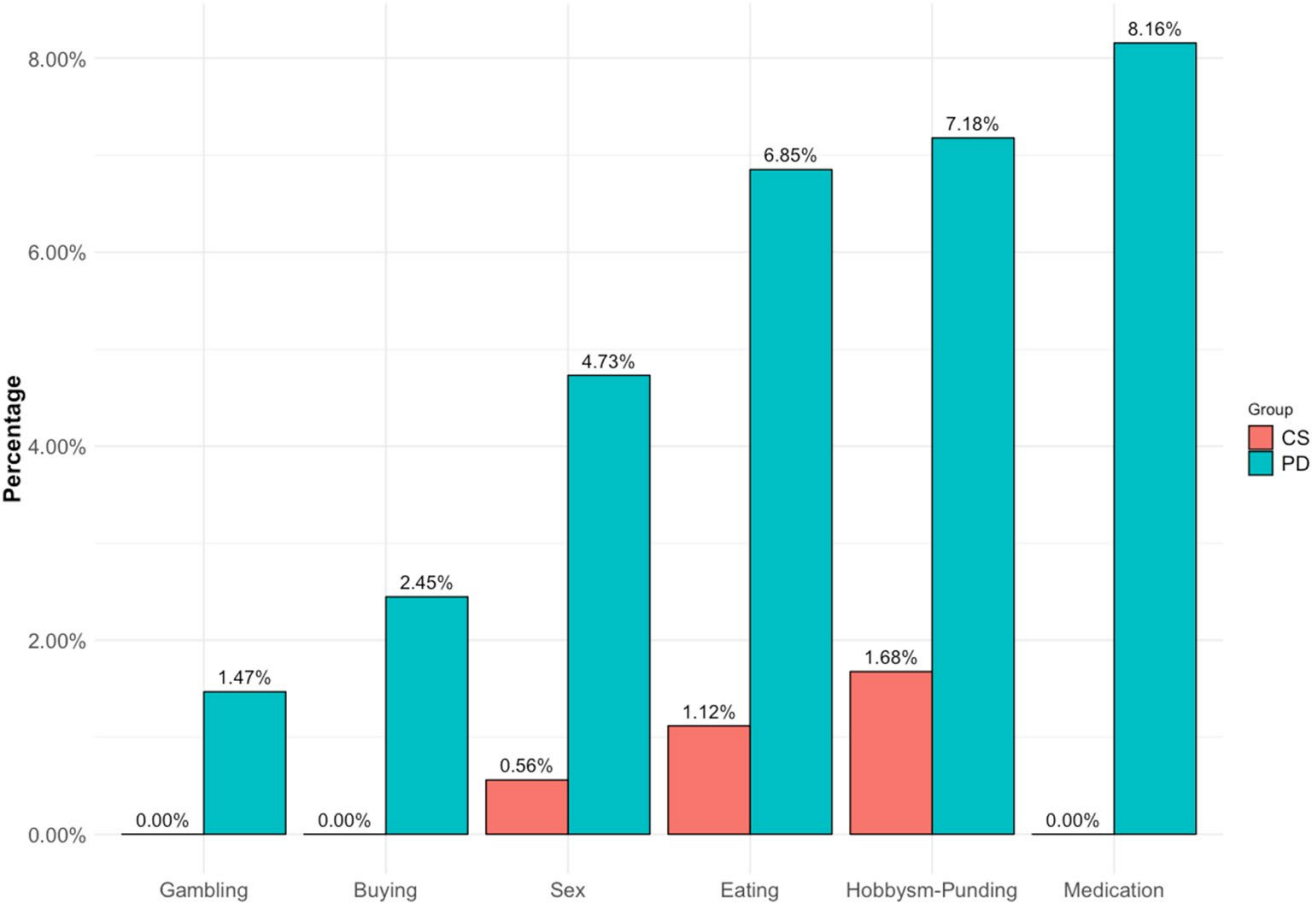
Distribution of impulse control disorders and compulsive behaviours in both groups.* CS *control subjects, *PD* Parkinson's disease patients.

Within the COPPADIS sample, 613 patients with PD and 179 CS completed the ICDs and CBs evaluation. ICDs and CBs were more frequent in patients with PD compared with the CS (ICDs 12.7% vs. 1.6%, respectively, *p* < 0.001; CBs 7.18% vs. 1.67%, respectively, *p* = 0.01). The most frequent ICD was compulsive eating in both groups and hobbysm-punding in terms of CBs (Fig. 1).

The analysis split by sex showed a higher presence of hypersexuality in male patients with PD (4.5% vs. 0.16%, *p* < 0.001). In the CS, the presence of hypersexuality was higher in the male participants, but it was not significant (0.55% vs. 0%, *p* = 0.85).

### Demographic characteristics of the participants with PD and CS

The demographic characteristics of the sample are shown in Table 1. Patients with PD were older than the CS (62.47 ± 9.09 vs. 60.84 ± 8.33 years, respectively; *p* = 0.004) and were more often men (59.87% vs. 47.48%, respectively; *p* = 0.004). Premorbid impulsive personality (8.65% vs. 1.1%, respectively; *p* < 0.001) and previous ICDs (16.1% vs. 0%, respectively; *p* < 0.001) were more frequent among patients with PD than CS, in addition to them having a higher rate of antidepressant treatment (25.12% vs. 11.73%, respectively; *p* < 0.001).

**Table 1:** Demographic characteristics of Parkinson’s disease and control subjects.

	Control subjects	Parkinson’s disease	*p* value
N	179	613	–
**Demographic**			
Sex (M/F)	85/94	367/246	**0.004**
Age (mean ± SD)	60.84 ± 8.33	62.47 ± 9.09	**0.004**
Culture level^a^	55/63/61	259/187/166	**0.02**
Lifestyle^b^	132/11/8/28	487/31/28/66	0.29
Habitat^c^	19/40/120	73/112/426	0.46
Premorbidity			
Historical ICDs or CBs	0 (0%)	99 (16.1%)	**< 0.001**
Premorbid impulsive personality	2 (1.1%)	53 (8.65%)	**< 0.001**
Family history of ICDs or CBs	4 (2.2%)	54 (8.8%)	0.92
Family history of PD	30 (16.75%)	170 (27.7%)	**0.004**
**Comorbidity**			
Antidepressant treatment	21(11.73%)	154(25.12%)	**< 0.001**
Anxiolytic treatment	22 (12.29%)	100 (16.31%)	0.23
Antipsychotic treatment	0 (0%)	13 (2.12%)	0.1
Smoke	79 (44.13%)	233 (38%)	0.16
Alcohol intake	36 (20.11%)	127(20.72%)	0.95

Results for univariate tests: chi-square test, *t* test or Wilcoxon test.

Significant *p* values are in bold.

*ICDs* impulse control disorders, *CBs* compulsive behaviours, *N* number of subjects, *M* male, *F* female, *SD* standard deviation.

^a^Culture level (Elementary/Hihg School/University education).

^b^Lifestyle (Married/Married with children/Others/Alone).

^c^Habitat (Rural/Semiurban/Urban).

### Parkinson’s disease features according to ICDs

In the PD group, those with ICDs had a more frequent previous history of ICDs or CBs (17.95% vs. 7.23%; *p* = 0.01) and premorbid impulsive personality (17.95% vs. 6.6%; *p* = 0.002) (Table 2).

**Table 2:** Demographic characteristics of Parkinson’s disease group according to the presence of impulse control disorders.

	ICDs negative	ICDs positive	*p* value
**Demographic**			
Sex (M/F)	318/217	49/29	0.65
Age (mean ± SD)	62.9 ± 8.92	59.46 ± 9.7	**0.002**
Culture level^a^	226/160/148	33/27/18	0.6
Lifestyle^b^	425/28/23/58	62/3/5/8	0.81
Habitat^c^	66/95/372	7/17/54	0.53
**Premorbidity**			
Historical ICDs or CBs	39 (7.23%)	14 (17.95%)	**0.01**
Premorbid impulsive personality	41 (6.6%)	14 (17.95%)	**0.002**
Familiar history of ICDs or CBs	48 (%)	6 (%)	0.87
**Comorbidity**			
Antidepressant treatment	129 (24.1%)	25 (32%)	0.12*
Anxiolytic treatment	90 (16.8%)	10 (12.82%)	0.43*
Antipsychotic treatment	10 (1.87%)	3 (3.8%)	0.62
Smoking	200 (37.4%)	33 (42.3%)	0.48
Alcohol intake	113 (21.12%)	14 (17.9%)	0.51

Results for univariate tests: chi-square test, *t* test or Wilcoxon test.

Significant *p* values are in bold.

*ICDs* impulse control disorders, *CBs* compulsive behaviours, *N* number of subjects, *M* male, *F* female, *SD* standard deviation.

*Results for multivariate analysis: multivariate linear regression or multivariate logistic regression adjusted by age, years of disease duration.

*Adjusted by age, years of disease evolution.

^a^Culture level (Elementary/Hihg School/University education).

^b^Lifestyle (Married/Married with children/Others/Alone).

^c^Habitat (Rural/Semiurban/Urban).

PD patients with ICDs were younger at evaluation (59.46 ± 9.7 vs. 62.9 ± 8.92; *p* = 0.002) and at disease onset (54.28 ± 9.88 vs. 58.42 ± 9.43; *p* < 0.001) (Table 3).

**Table 3:** Motor and non motor evaluations in Parkinson’s disease according to the presence of impulse control disorders.

		ICDs negative	ICDs positive	*p* value
Number of subjects		535	78	–
Family history PD	N	145 (27.1)	25 (32.05)	0.43
Age at onset	Age (mean ± SD)	58.42 ± 9.43	54.28 ± 9.88	**< 0.001**
Disease progression time	Years (mean ± SD)	5.42 ± 4.51	6.25 ± 3.87	**0.008**
UPDRS score (mean ± SD)	II OFF	2.66 ± 1.79	3.34 ± 2.27	0.009*
	II ON	1.69 ± 1.80	1.79 ± 1.99	0.72*
	III OFF	22.4 ± 10.97	24.52 ± 12.75	0.22*
	III ON	16.18 ± 8.27	15.86 ± 7.74	0.98*
	IV	1.91 ± 2.37	2.48 ± 2.42	0.19*
Phenotype (undetermined/PIGD/Tremor)	N	80/205/248	17/33/28	0.13
Dominant hemibody	N	30/1/149/89/166/100	2/0/24/14/24/14	0.82
Motor fluctuations	N (%)	169 (31.56)	29 (37.18)	0.75*
Dyskinesias	N (%)	92 (17.19)	14 (17.95)	0.68*
NMSS	Score (mean ± SD)	41.33 ± 33.77	60.37 ± 42.48	< 0.001*
PDSS	Score (mean ± SD)	116.83 ± 25.63	102.58 ± 25.84	< 0.001*
REM sleep behaviour disorder	N (%)	198 (37)	35 (44.87)	0.19
BECK (depression yes)	N (%)	262 (48.97)	51 (65.38)	0.004*
BECK score	(mean ± SD)	8.24 ± 7.02	12.46 ± 8.51	< 0.001*
BECK-depression inventory	Score (mean ± SD)	8.25 ± 7.02	12.47 ± 8.51	< 0.001*
	Subclinic/minor/major	79/89/94	19/16/16	0.38
	No depression vs subclinic	273/79	27/19	0.007*
	No depression vs minor	273/89	27/16	0.02*
	No depression versus major	273/94	27/16	0.11*
FOG	Score (mean ± SD)	3.38 ± 4.23	5.73 ± 5.73	< 0.001*
MMSE	Score (mean ± SD)	29.21 ± 1	29.15 ± 1.1	0.22*
PD-CRS	Score (mean ± SD)	91.64 ± 15.67	92.56 ± 17.44	0.36*
	Non CI/MCI/dementia	374/151/3	55/22/1	0.76*
	Fronto-subcortical	63.74 ± 14.39	64.93 ± 15.88	0.49*
	Posterior cortical	27.89 ± 2.99	27.63 ± 2.89	0.16*
NPI (severity × frequency)	Score (mean ± SD)	5.48 ± 7.32	8.6 ± 10.28	0.005*
PDQ-39	Score (mean ± SD)	15.64 ± 12.36	23.38 ± 14.93	< 0.001*
WHOQOL-8	Score (mean ± SD)	7.33 ± 1.58	6.97 ± 1.61	0.11

Results for univariate test:chi-square test, *t* test or Wilconxon test.

Significant *p* values are in bold.

*ICDs* impulse control disorders, *FOG* Freezing of Gait Scale, *UPDRS* Unified Parkinson’s Disease Rating Scale, *N* number of subjects, *NMSS* Non Motor Symptoms Scale, *NPI* Neuropsychiatry Inventory, *PD* Parkinson’s disease, *PD-CRS* Parkinson’s Disease Cognitive Rating Scale, *PDQ-39* 39 items Parkinson’s Disease Questionnaire, *PDSS* Parkinson’s Disease Sleep Scale, *REM* rapid eye movements, *SD* standart deviation, *WHOQOL-8* 8 items World Health Organization Quality of Life.

*Results for multivariate analysis: multivariate linear regression or multivariate logistic regression adjusted by age, age at onset and years of disease evolution.

**Figure 2:**
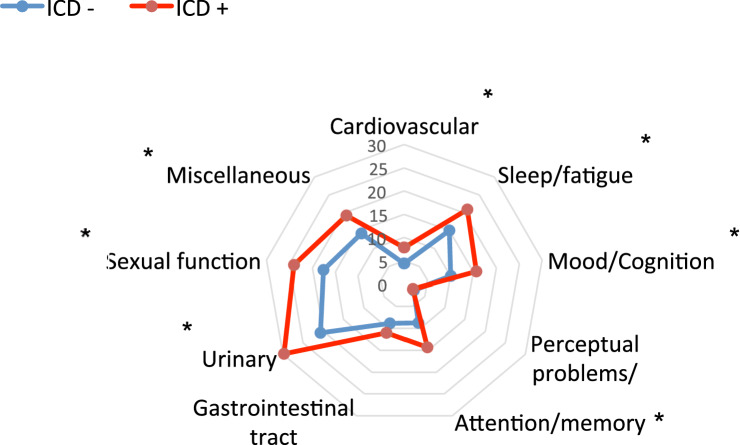
Mean nonmotor symptoms scale domain score in patients with Parkinson's disease according to the presence of impulse control disorders. Multivariate linear regression adjusted by age, age at disease onset and years of disease evolution. *Statistically significant (*p* < 0.05). *ICD* impulse control disorders.

Concerning the PD features according to the presence of ICDs, the motor characteristics were similar in both groups, except for a higher score in the freezing of gait (FOG) scale in patients with ICDs (5.73 ± 5.73 vs. 3.38 ± 4.23; *p* < 0.001) (Table 3). However, the differences in nonmotor features as well as in the performance and quality of daily living activities were notable. The score on the Non-Motor Symptoms Scale (NMSS) was higher in patients with ICDs (60.37 ± 42.48 vs. 41.33 ± 33.77; *p* < 0.001). In addition, when the NMSS subdomains were analysed, the cardiovascular, sleep/fatigue, mood/cognition, attention/memory, urinary, sexual and miscellaneous domains had a higher impact on the patients with ICDs (Fig. 2). Along these lines, the Neuropsychiatry Inventory (NPI) showed that, overall, patients with ICDs presented with a more prominent burden of these symptoms (8.6 ± 10.28 vs. 5.48 ± 7.32; *p* = 0.005). In terms of mood, patients with ICDs showed higher rates of depression (48.97% vs 65.38%, *p* = 0.004). Analyzing the subtype of depression according to the Beck Depression Inventory, patients with ICDs showed a higher ratio of subclinic and minor depression compared to those without ICDs. Anxiety, euphoria and irritability were the features specifically associated with patients with ICDs.

Apart from the NMSS sleep score, the Parkinson’s Disease Sleep Scale showed that patients with ICDs presented with poorer sleep quality, as shown by a lower score (102.58 ± 25.84 vs 116.83 ± 25.63; *p* < 0.001).

The cognitive status did not differ between groups. After assessing the separate domains of the Parkinson’s Disease Cognitive Rating Scale (PD-CRS), we did not find differences between the patients with and without ICDs.

**Figure 3:**
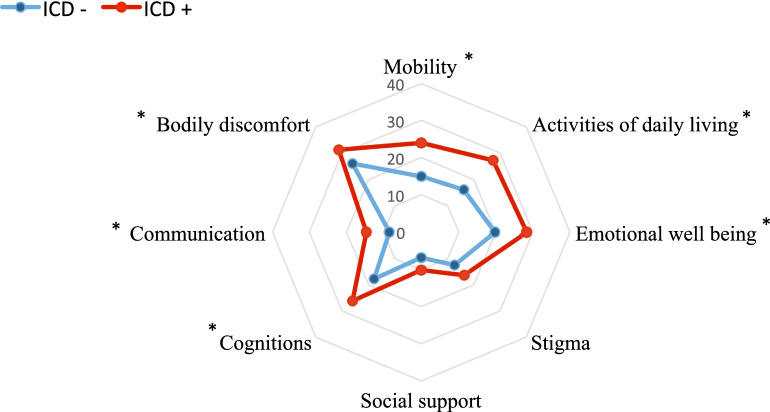
39 items Parkinson's disease Questionaire (PDQ-39) domains in Parkinson's disease patients according to the presence of impulse control disorders. Score expressed by a percentage (0–100%). Multivariate linear regression adjusted by age, age at disease onset and years of disease evolution. *Statistically significant (*p* < 0.05). *ICD* impulse control disorders.

The quality of life as measured by the 39-item Parkinson’s disease Questionnaire (PDQ-39) was poorer in patients with ICDs (23.38 ± 14.93 vs. 15.64 ± 12.36; *p* < 0.001). Analysing the domains of the PDQ-39, ICDs had a higher impact on patients in terms of mobility, activities of daily living, body discomfort, communication, cognition and emotional well-being (Fig. 3). ICDs were also associated with a poorer score on the performance of daily activities, as shown by a higher motor items score in the Unified Parkinson's Disease Rating Scale (UPDRS) part II in off condition (2.66 ± 1.79 vs. 3.34 ± 2.27; *p* = 0.009) (Table 3).

In terms of CBs, the results were similar to those observed for the ICDs (Supplementary Material Table 1). There were few exceptions for the age, age at onset, disease progression time and the score of the UPDRS part II in off that were similar in the group of CBs positive and negative.

Regarding dopaminergic treatment, we analysed patients receiving DA treatment, levodopa and/or monoamine oxidase-B inhibitors. After controlling for levodopa-equivalent dose, we found that ICDs were more frequent in patients on DA treatment and the agonist dose was related to the presence of ICDs (237.65 ± 194.72 vs. 174.72 ± 164.92; *p* = 0.02) (Table 4).

**Table 4:** Dopaminergic replacement treatment and its relationship with impulse control disorders.

		ICDs negative	ICDs positive	P value
Dopaminergic agonists	N (%)	355 (66.35%)	67 (85.9%)	0.006*
L-DOPA	N (%)	381 (71.2%)	60 (76.9%)	0.59
MAO-B inhibitors	N (%)	384 (71.7%)	64 (82%)	0.18
DA equivalent dose	Mean ± SD	174.72 ± 164.92	237.65 ± 194.72	0.02*
LDOPA daily dose	Mean ± SD	339.3 ± 307.9	388.01 ± 310.12	0.57
LDOPA equivalent daily dose	Mean ± SD	539.02 ± 405.63	636.99 ± 450.35	0.25
Time on LDOPA	Months (mean ± SD)	32.99 ± 44.49	39.23 ± 41.79	0.91
Time on agonists	Months (mean ± SD)	33.62 ± 41.95	45.86 ± 42.34	0.14

Results for univariate tests: chi-square test, *t* test or Wilcoxon test.

*ICDs* impulse control disorders, *N* number of subjects, *DA* dopamine agonists, *SD* standard deviation.

*Results for multivariate analysis: multivariate linear regression or multivariate logistic regression adjusted by age, age at onset, years of disease progression.

To analyse the influence of the type of DA, we compared not taking any agonists with the risk linked to each type of agonist. For this analysis, we observed that Rotigotine had the lowest risk (OR 2.45; 95% CI 1.06–5.8; *p* = 0.03) and Ropirinole the highest (OR 2.82; 95% CI 1.25–6.57; *p* = 0.013) (Table 5). Nevertheless, the analysis of ICDs risk among agonists did not show statistically significant differences.

**Table 5:** Comparison among patients on different agonists with those without them and the relationship to impulse control disorders.

	N	ICDs	*P* value
Pramipexole	208	32 (15.4%)	*p* = 0.017 (OR 2.47; CI 95% 1.2–5.42)
Ropinirole	107	18 (16.8%)	*p* = 0.013 (OR 2.82; CI 95% 1.25–6.57)
Rotigotine	99	15 (15.5%)	*p* = 0.03 (OR 2.45; CI 95% 1.06–5.8)

Results for multivariate logistic regression adjusted by age, age onset and years of disease.

*ICDs* impulse control disorders, *N* number of subjetcs, *OR* odds ratio, *CI* confidence interval.

**Figure 4:**
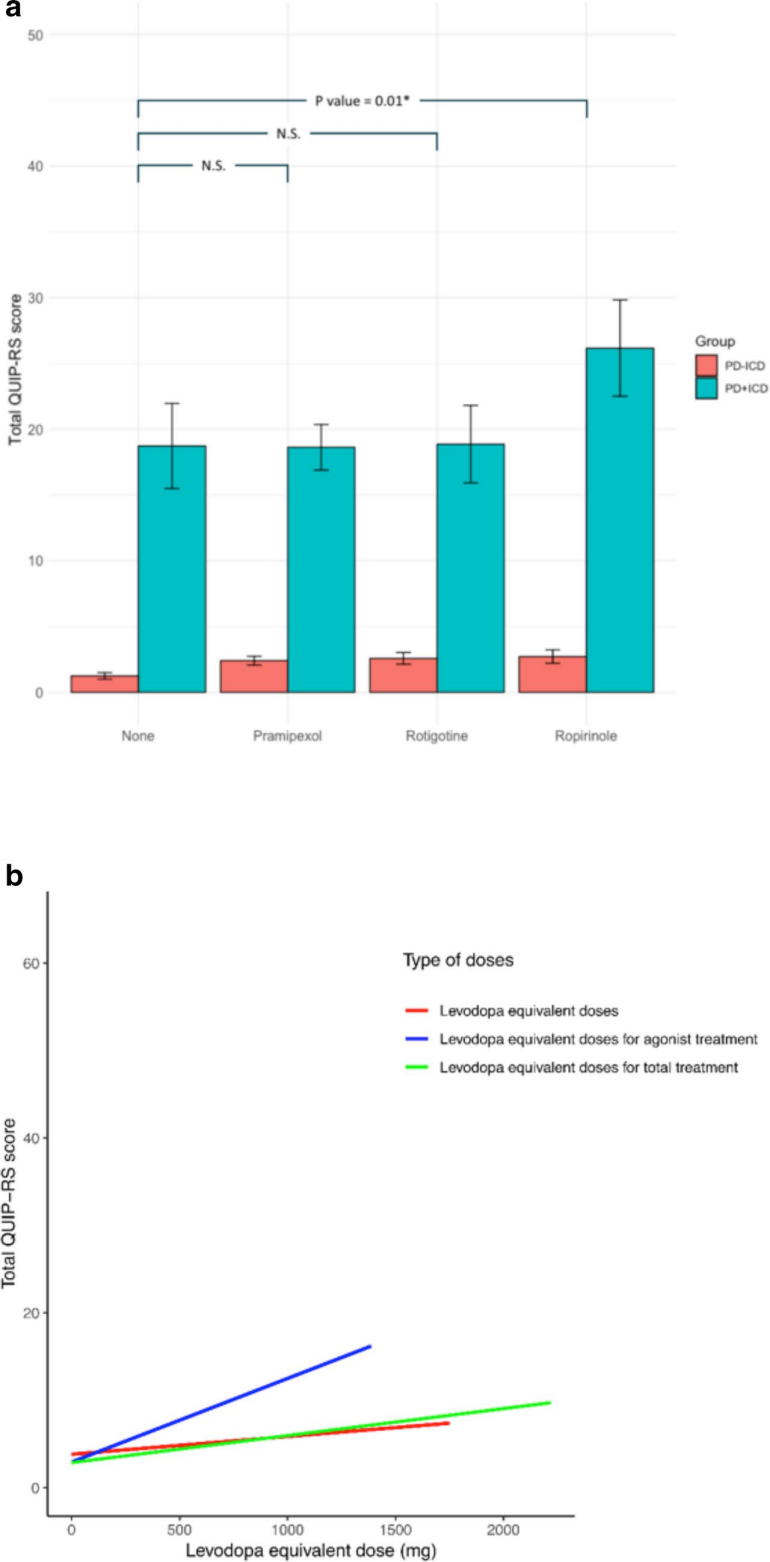
(**a**) Relationship between severity of impulse control disorders and type of dopamine agonist in patients with impulse control disorders positive (PC + ICD) and negative (PD-ICD). *NS* Not significant (**b**) Relationship between severity of impulse control disorders and dopaminergic treatment dose.

To examine the influence of ICDs severity according to treatment, we analysed the QUIP-RS total score with dopaminergic treatment characteristics. As expected, we observed that patients with PD undergoing DA treatment had a significantly greater QUIP-RS total score compared with the patients with PD without agonist treatment, controlling for age, age at disease onset and disease progression time (5.47 ± 9.24 vs. 2.26 ± 5.78, respectively; F = 19.39; *p* < 0.001). When we compared the type of DA with the non-use of DA and the severity of ICDs/CBs using the QUIP-RS total score, Ropinirole was associated with the greatest significant increase in severity (Beta = 4.40; 95% CI 2.44–6.35; F = 7.411; *p* < 0.001). This result remained significant after correcting for confounding variables such as age, age at disease onset and disease progression time (Beta = 3.83; 95% CI 1.62–6.04; F = 4.773; *p* < 0.001). Moreover, the increased QUIP-RS total score in patients with ICDs undergoing Ropinirole treatment was significant (Beta = 5.97; 95% CI 1.51–10.43; F = 105.6; *p* < 0.001), but not for patients with other types of DA (Fig. 4a). The results were the same when correcting for confounding variables (age, age at disease onset and disease progression time )(Beta = 5.99; 95% CI 1.38–10.59; F = 62.35; *p* < 0.001). On the other hand, we observed that the QUIP-RS total score was significantly increased according to DA equivalent dose (Beta = 0.853; 95% CI 0.41–1.30; F = 9.02; *p* < 0.001) and daily levodopa equivalent dose (Beta = 0.274; 95% CI 0.07–0.47; F = 6.89; *p* < 0.01), but not with levodopa dose alone (Fig. 4b).

For the subanalysis of the group with an age at onset younger than 50 years, this group presented with a higher rate of ICDs (19.78%) and differences on the cultural level. Other features such as a premorbid impulsive personality, non-motor symptoms according to NMSS and BDI and poorer quality of life were similar to the entire sample. In the same line with the global analysis, a higher rate in the FOG scale was shown in the group under 50 years old (Supplementary Material, Tables 4 and 5). In the DA treatment group, the results were also similar except for poorer motor performance in off in patients with ICDs (22.31 ± 10.43 vs. 25.46 ± 13.16; *p* = 0.02) (Supplementary Material, Tables 2 and 3).

## Discussion

The objective of the present study was to assess ICDs and CBs in patients with PD and CS, as well as the clinical features linked to these disorders in both groups. We found that in the patients with PD, ICDs and CBs were more frequent compared with the CS (ICDs 12.7% vs. 1.6%, respectively; *p* < 0.001; CBs 7.18% vs. 1.67%, respectively; *p* = 0.01). The proportion of ICDs found in our sample was similar to that reported in the DOMINION study^2^, which is the largest PD prevalence study to date, in which they found ICDs in 13.2% of cases, in accordance with our study and with other European works^17^. This result is in contrast to the high rate of ICDs found in some other European studies, including in the Spanish population^3,10,11^. The prospective Italian study, ICARUS, had found a rate of ICDs at the basal evaluation of 29.3%, which was higher than in our baseline assessment in COPPADIS^3^. These discrepancies could partially be explained by the sociocultural differences in both groups and by the differing tools used for ICDs screening. In the ICARUS study, the Minnesota Impulsive Disorders Interview (MIDI) and QUIP were used, whereas in COPPADIS, the QUIP-RS was applied. Following the MDS recommendations, QUIP-RS has more accurate clinimetric properties for application in PD, being “recommended,” whereas MIDI lacks some criteria validity when compared with the QUIP-RS^18^. In addition, ICDs screening using QUIP could overestimate ICDs frequency since Papay et al. found that, up to 40% of patients who scored positive for ICDs on the QUIP did not fulfil the diagnosis of ICDs^19^. However, the QUIP-RS used in COPPADIS has stricter ICDs diagnostic criteria; thus, the proportion found in our study could be more accurate for the identification of ICDs.

On the other hand and in contrast to two Spanish studies, Vela et al. had found an ICDs estimation that reached 58.3% in their cohort of early-onset PD; and in their study of patients undergoing DA treatment, García-Ruiz et al. found this rate increased up to 39.1%^10,11^. In COPPADIS, when we analysed these two groups separately, the prevalence of ICDs reached 15.87% in patients with PD undergoing DA treatment and 19.78% in patients with an age at disease onset younger than 50 years. Again, these differences could be justified by the different estimation of ICDs provided by QUIP as screening tool that could overestimate the prevalence of ICDs^19^.

Accounting for the features in PD and CS, we observed that a previous history of ICDs as well as premorbid impulsive personality were more frequent in patients with PD than in CS. A higher rate of antidepressant treatment was also shown in patients with PD, which could be an intrinsic feature of PD, given a higher burden of nonmotor symptoms is present compared with CS, and consequently, depression is also more frequent^7,8^.

In the PD group, as previous studies have reported, those who had ICDs were younger and their disease onset was earlier^2, 3,10,11,20^. On the other hand, and in contrast to previous studies, in our cohort, ICDs were not related to a longer disease duration, nor a higher exposure to DRT^3,21,22^. This result is interesting, given previous studies had hypothesised that the presence of ICDs in young patients could be explained by a dysfunction of the mesocorticolimbic loop due to the influence of the DRT on a relatively preserved ventral striatum^23^. However, recent studies have supported the contrary version given that, reduced dopamine synthesis capacity in the nucleus accumbens as well as reduced functional connectivity in this area have been shown^9,24^. Consequently, the higher rate of ICDs in young patients could not be fully explained by the neurodegenerative pattern progression. Genetic factors could instead be involved in the early disease onset and ICDs in these patients^5,25^.

In patients with PD who have ICDs, a premorbid history of ICDs and impulsive personality were more frequent, according to observations in previous studies developed in European countries and worldwide^2,17,26^.

Concerning motor performance, in our case and according to the vast majority of studies, the motor status did not differ in patients with ICDs^3,10,11^. However, it is interesting to note that, although the motor performance according to the UPDRS III was not different according to the presence of ICDs, the scores on the FOG scale were higher in patients with ICDs. This observation was similar in the subgroup of early disease onset, suggesting a shared physiopathological background or due to the perception of the patients since a poorer status was also found in the motor subdomain of the PDQ-39 in patients with ICDs.

The neuropsychiatric sphere is highly impaired in patients with ICDs, as is shown in multiple studies^3,14^. In COPPADIS, we found a high rate of depression as well as a poorer perception of mood disturbances according to the NMSS, PDQ-39 and NPI. The link between mood and impulsivity is not fully understood because it is not clear whether there is a predisposing factor or whether it is simply a consequence of the ICDs^6–8^. Indeed, it is difficult to elucidate by the comorbidity of both disorders. However, we must take into account that there are genetic forms of PD with prominent nonmotor manifestations, and a common genetic background could therefore underlie both, mood disorders and ICDs^27–29^. In COPPADIS, depression was more frequent in patients with PD compared with CS, which could suggest it as an intrinsic feature of PD rather than a consequence of ICDs. On the other hand, and also correlated with mood disorders, sleep disturbances are usually present in patients with ICDs^30–32^. The relationship could again be bidirectional, as in depression, but it also appears that there is a shared neurobiological substrate, given some genetic risk factors might justify the coexistence of both ICDs and sleep problems^33^. On the other hand, the intake of antidepressant drugs was not different between patients with ICDs positive or negative in the whole cohort. This observation is important since it could point out to a infradiagnosis of depression in patients with ICDs.

Apart from the impact of neuropsychiatric symptoms and sleep, in line with previous studies^3^, other nonmotor symptoms were more prominent in patients with ICDs. Urinary and cardiovascular symptoms were more frequent in patients with ICDs, in agreement with the Italian study ICARUS and other series. It is interesting to note that these patients present with a high burden of nonmotor symptoms from different spheres; thus, we should keep this into mind when performing an integral assessment in this particular group of individuals.

Concerning cognitive performance related to ICDs, we did not find any difference between the groups, even when analysing the subgroups of patients undergoing DA treatment or early-onset PD. It contrasts with the abnormalities noted in other studies in which executive dysfunction as well as impairment in the reward-related decision process and set shifting have been noticed^13,14,34^. In COPPADIS, the global cognition did not differ between groups. Analysing the frontal-subcortical domains of PD-CRS separately, no differences were found. To better understand the reasons for these discrepancies, future neuroimaging studies would be useful to determine the functionality of this area in our cohort.

Regarding the DRT and its influence on ICDs, we found that DA use had greater impact on ICDs; it was dose-dependent and related to severity, which was in line with the observation noted in multiple studies across populations^2,10,11,35^. However, the risks linked to the type of agonist can be different. We found, according to another Spanish study and a European multicentre study, that Rotigotine had a low risk of developing ICDs, and Ropirinole the highest^11,36^. As Rizos et al. explained, the association between Rotigotine and ICDs is poorly understood, but it could be associated with its mechanism of action from continuous dopaminergic stimulation^37^. This possibility is important, given we could consider this in patients with other risk factors for ICDs in the case that DA treatment is required.

On the other hand, it is interesting to highlight the fact that longer levodopa or DA exposure was not related to the appearance of ICDs; thus, ICDs could appear at any time in the disease course. Therefore, clinicians should be aware of and investigate ICDs over time.

This study has some limitations. The presented data correspond to the cross-sectional evaluation at baseline of COPPADIS cohort, nevertheless, the assessment of the ICDs/CBs behaviour over time would be more informative. This issue would be overcome in the future since the follow up of this cohort is on-going and we will obtain the longitudinal data^38^. On the other hand, following the methodology of the study, the UPDRS part II evaluation only included motor items to assess the phenotype (tremor dominant, postural instability and gait disorders or undetermined). It would be more accurate to evaluate the whole UPDRS part II to establish the handicaps in the performance of the daily activities according this scale. To partially compensate this issue, we assessed the PDQ-39 that also included activities of daily living.

## Conclusions

ICDs and CBs are common nonmotor features in patients with PD compared with CS. Some premorbid features such as a previous impulsive personality act as risk factors for developing these disorders. DRT had a large impact on ICDs, and DAs were the drugs with the greatest effect. However, patients with ICDs had a high burden of nonmotor symptoms belonged to mood, cardio-vascular, sleep, urinary domains among others as well as a poor quality of life. Hence, apart from treatment, patients might have a genetic background that could influence the presence of a prominent non-motor phenotype.

## Supplementary information

Supplementary Information.
