# Supplementary material for: Non-motor symptom burden in patients with Parkinson’s disease with impulse control disorders and compulsive behaviours: results from the COPPADIS cohort
Source: Sci Rep. 2020 Oct 9;10:16893. doi: 10.1038/s41598-020-73756-z (PMC7547680; doi:10.1038/s41598-020-73756-z)
Supplement: Supplementary file 1 — Supplementary Information. [file 41598_2020_73756_MOESM1_ESM.docx]

**SUPPLEMENTARY MATERIAL.**

Non-Motor Symptom Burden in Patients with Parkinson’s Disease with Impulse Control Disorders and Compulsive Behaviours: Results from the COPPADIS Cohort

Jesús S, MD, PhD^1,2^; Labrador-Espinosa MA, MSc^1,2^; Adarmes AD, MD^1,2^; Méndez Del Barrio C, MD^1,2^; Martínez-Castrillo JC, MD, PhD^3^; Alonso-Cánovas A, MD, PhD^3^; Sánchez Alonso P, MD^4^; Novo-Ponte S, MD^4^; Alonso-Losada MG, MD^5^; López Ariztegui N, MD^6^; Segundo Rodríguez JC, MD^6^; Morales MI, MD^6^; Gastón I, MD^7^; Lacruz Bescos F, MD^7^; Clavero Ibarra P, MD^7^; Kulisevsky J, MD, PhD^2,8^; Pagonabarraga J, MD, PhD^2^, ^8^; Pascual-Sedano B, MD^2,8,11^; Martínez-Martín P, MD, PhD^2,9^; Santos-García D, MD, PhD^10^; Mir P, MD, PhD^1,2*^; COPPADIS Study Group.

Table 1. Motor and non motor evaluations in Parkinson’s disease according to the presence of compulsive behaviours.

|  |  | CBs negative | CBs positive | P value |
| --- | --- | --- | --- | --- |
| Number of subjects | N | 569 | 44 | - |
| Demographic | Sex (M/F) | 340/229 | 27/17 | 0.95 |
|  | Age (Mean ± SD) | 62.56 ± 9.08 | 61.30 ± 9.24 | 0.31 |
| Family history PD | N | 157 (27.59) | 13 (29.54) | 0.92 |
| Age at onset | Age (Mean ± SD) | 58.07 ± 9.52 | 55.50 ± 10.14 | 0.07 |
| Disease progression time | Years (Mean ± SD) | 5.50 ± 4.45 | 5.88 ± 4.39 | 0.39 |
| UPDRS  Score (Mean ± SD) | II OFF | 2.71 ± 1.86 | 3.24 ± 2.02 | 0.13 |
|  | II ON | 1.72 ± 1.82 | 1.65 ± 2.01 | 0.77* |
|  | III OFF | 22.34 ± 11.11 | 27.00 ± 12.08 | 0.05* |
|  | III ON | 16.11 ± 8.27 | 16.28 ± 7.79 | 0.97* |
|  | IV | 1.92 ± 2.36 | 2.72 ± 2.49 | **0.04*** |
| Phenotype (undetermined/PIGD/Tremor) | N | 89/220/258 | 8/18/18 | 0.82 |
| Dominant hemibody | N | 30/1/160/99/174/105 | 2/0/13/4/16/9 | 0.68 |
| Motor fluctuations | N (%) | 179 (31.45) | 19 (43.18) | 0.15* |
| Dyskinesias | N (%) | 95 (16.69) | 11 (25) | 0.23* |
| NMSS | Score (Mean ± SD) | 42.46 ± 34.84 | 60.50 ± 40.36 | **0.003*** |
| PDSS | Score (Mean ± SD) | 115.95 ± 25.92 | 102.76 ± 25.20 | **0.004*** |
| Pain | N (%) | 330 (57.99) | 23 (52.27) | 0.43* |
| REM sleep behaviour disorder | N (%) | 217 (38.14) | 16 (36.36) | 0.83* |
| BECK (depression yes) | N (%) | 282 (49.56) | 31 (70.45) | **0.007*** |
| BECK- Depression Inventory | Score (Mean ± SD) | 8.49 ± 7.24 | 12.69 ± 7.76 | **<0.001*** |
|  | Subclinic/minor/major | 87/99/96 | 11/6/14 | 0.88* |
|  | No depression vs subclinic | 287/87 | 27/11 | 0.42* |
|  | No depression vs minor | 287/99 | 27/6 | **0.002*** |
|  | No depression vs major | 287/96 | 27/14 | 0.13* |
| FOG | Score (Mean ± SD) | 3.53 ± 4.45 | 5.39 ± 5.01 | **0.009*** |
| MMSE | Score (Mean ± SD) | 29.20 ± 1 | 29.18 ± 1.09 | 0.97* |
| PD-CRS | Score (Mean ± SD) | 91.74 ± 15.89 | 91.95 ± 16.19 | 0.85* |
|  | Non CI/MCI/dementia | 397/164/3 | 32/9/1 | 0.22 |
|  | Fronto-subcortical | 63.87 ± 14.63 | 64.23 ± 14.06 | 0.82* |
|  | Posterior cortical | 27.89 ± 2.95 | 27.71 ± 3.27 | 0.89* |
| NPI (severity x frequency) | Score (Mean ± SD) | 5.67 ±7.76 | 9.17 ± 8.43 | **0.02*** |
| PDQ-39 | Score (Mean ± SD) | 15.99 ± 12.54 | 24.85 ± 15.45 | **<0.001*** |
| WHOQOL-8 | Score (Mean ± SD) | 7.31 ± 1.59 | 7.06 ± 1.46 | 0.625 |
| Results for univariate tests: chi-square test, t-test or Wilcoxon test.  *Results for multivariate analysis: multivariate linear regression or multivariate logistic regression adjusted by age, years of disease duration.  CBs: compulsive behaviours, FOG: Freezing of Gait Scale, UPDRS: Unified Parkinson’s Disease Rating Scale, N: number of subjects, NMSS: Non Motor Symptoms Scale, NPI: Neuropsychiatry Inventory, PD: Parkinson’s disease, PD-CRS: Parkinson’s Disease Cognitive Rating Scale, PDQ-39: 39 items Parkinson’s Disease Questionnaire, PDSS: Parkinson’s Disease Sleep Scale, REM: Rapid Eye Movements, SD: standard deviation, WHOQOL-8: 8 items World Health Organization Quality of Life. | | | | |

Table 2. Demographic characteristics of Parkinson’s disease according to the presence of impulse control disorders in patients on dopaminergic agonist treatment.

|  |  | **ICDs negative** | **ICDs positive** | **P Value** |
| --- | --- | --- | --- | --- |
|  | N | 355 | 67 | - |
| Demographic | Sex (M/F) | 220/135 | 45/22 | 0.5 |
|  | Age (Mean ± SD) | 62.24±8.7 | 59.34±9.63 | **0.02** |
|  | Culture level^1^ | 146/108/100 | 26/24/17 | 0.68 |
|  | Lifestyle^2^ | 290/15/15/34 | 51/1/5/7 | 0.49 |
|  | Habitat^3^ | 43/56/255 | 6/15/46 | 0.36 |
| Premorbidity | Historical ICDs or CBs | 47 (41.59) | 20 (74.07) | **0.001** |
|  | Premorbid impulsive personality | 25 (7.04) | 11 (16.41) | **0.023** |
|  | Family history of ICDs or CBs | 24 (6.76) | 6 (8.95) | 0.7 |
|  | Family history of PD | 93 (26.19) | 21 (31.34) | 0.47 |
| Comorbidity | Antidepressant treatment | 68 (19.15) | 23 (34.32) | **0.012*** |
|  | Anxiolytic treatment | 44 (12.39) | 10 (14.92) | 0.51* |
|  | Antipsychotic treatment | 5 (1.40) | 3 (4.47) | 0.17* |
|  | Smoke | 129 (36.33) | 28 (41.79) | 0.48 |
|  | Alcohol intake | 75 (21.12) | 13 (18.40) | 0.87 |
| Results for univariate tests: chi-square test, t-test or Wilcoxon test.  *Results for multivariate analysis: multivariate linear regression or multivariate logistic regression adjusted by age, years of disease duration.  ^1^ Culture level (Elementary / High School / Universitary), ^2^ Life style (Married/Married with children/Others/Alone), ^3^ Habitat (Rural/Semiurban/Urban)  ICDs: impulse control disorders, CBs: compulsive behaviours, N: number of subjects, M: male, F: female, PD: Parkinson’s disease | | | | |
|  | | | | |

Table 3. Motor and non motor evaluations in Parkinson’s disease according to the presence of impulse control disorders in patients on dopaminergic agonist treatment.

|  |  | **ICDs negative** | **ICDs positive** | **P value** |
| --- | --- | --- | --- | --- |
| Number of subjects |  | 355 | 67 |  |
| Family history PD | N | 93 | 21 | 0.47 |
| Age at onset | Age (Mean ± SD) | 57±8.92 | 53.86±9.72 | **0.015** |
| Disease progression time | Years (Mean ± SD) | 6.07±4.71 | 6.57±3.97 | 0.1 |
| UPDRS  Score (Mean ± SD) | II OFF | 2.67±1.86 | 3.54±2.38 | **0.001*** |
|  | II ON | 1.67±1.82 | 1.9±2.1 | 0.55* |
|  | III OFF | 22.31±10.43 | 25.46±13.16 | **0.02*** |
|  | III ON | 15.59±8.13 | 15.56±8.05 | 0.77* |
|  | IV | 2.06±2.36 | 2.6±2.48 | 0.15* |
| Phenotype (undetermined/PIGD/Tremor) | N | 51/148/156 | 12/30/25 | 0.55 |
| Dominant hemibody | N | 24/102/52 | 2/19/12 | 0.79 |
| Motor fluctuations | N (%) | 130 (36.61) | 26 (38.80) | 0.94* |
| Dyskinesias | N (%) | 70 (19.71) | 12 (17.91) | 0.53* |
| NMSS | Score (Mean ± SD) | 38.57±30.52 | 59.51±43 | **<0.001*** |
| PDSS | Score (Mean ± SD) | 115.78±26.26 | 102.43±26.79 | **<0.001*** |
| Pain | N (%) | 200 (56.33) | 39 (58.2) | 0.94* |
| REM sleep behaviour disorder | N (%) | 125 (35.2) | 29 (43.2) | 0.2 |
| BECK (depression yes) | N (%) | 153 (43.1) | 45 (67.16) | **<0.001*** |
| BECK- Depression Inventory | Score (Mean ± SD) | 7.73±6.71 | 12.49±8.25 | **<0.001*** |
|  | Subclinic/minor/major | 39/53/61 | 16/14/15 | 0.71 |
|  | No depression vs subclinic | 202/39 | 22/16 | **0.001*** |
|  | No depression vs minor | 202/53 | 202/61 | **0.01*** |
|  | No depression vs major | 22/14 | 22/15 | **0.03*** |
| FOG | Score (Mean ± SD) | 3.6±4.52 | 6.18±5.94 | **<0.001*** |
| MMSE | Score (Mean ± SD) | 29.15±1.01 | 29.24±1.01 | 0.97* |
| PD-CRS | Score (Mean ± SD) | 92.02±15.81 | 93.8±16.81 | 0.8* |
|  | Non CI/MCI/dementia | 250/79/22 | 48/16/3 | 0.84 |
|  | Fronto-subcortical | 64.16±14.5 | 65.97±15.64 | 0.88* |
|  | Posterior cortical | 27.86±3.01 | 27.83±2.57 | 0.52* |
| NPI (severity x frequency) | Score (Mean ± SD) | 4.89±6.83 | 9.36±10.81 | **<0.001*** |
| PDQ-39 | Score (Mean ± SD) | 15.08±11.85 | 23.55±15.1 | **<0.001*** |
| WHOQOL-8 | Score (Mean ± SD) | 7.35±1.63 | 6.98±1.59 | 0.1* |
| Results for univariate tests: chi-square test, t-test or Wilcoxon test.  *Results for multivariate analysis: multivariate linear regression or multivariate logistic regression adjusted by age, years of disease duration.  ICDs: impulse control disorders, FOG: Freezing of Gait Scale, UPDRS: Unified Parkinson’s Disease Rating Scale, N: number of subjects, NMSS: Non Motor Symptoms Scale, NPI: Neuropsychiatry Inventory, PD: Parkinson’s disease, PD-CRS: Parkinson’s Disease Cognitive Rating Scale, PDQ-39: 39 items Parkinson’s Disease Questionnaire, PDSS: Parkinson’s Disease Sleep Scale, REM: Rapid Eye Movements, SD: standard deviation, WHOQOL-8: 8 items World Health Organization Quality of Life. | | | | |

Table 4. Demographic characteristics of Parkinson’s disease according to the presence of impulse control disorders in patients with an age at disease onset under 50 years old.

|  |  | **ICDs negative** | **ICDs positive** | **P value** |
| --- | --- | --- | --- | --- |
|  | N | 113 | 27 |  |
| Demographic | Sex (M/F) | 79/34 | 13/14 | 0.055 |
|  | Age (Mean ± SD) | 50.56 ± 7.46 | 49 ± 6.56 | 0.28 |
|  | Culture level^1^ | 35/30/48 | 5/16/6 | **0.004** |
|  | Lifestyle^2^ | 89/6/9/9 | 20/2/4/1 | 0.59 |
|  | Habitat^3^ | 11/18/84 | 1/5/21 | 0.59 |
| Premorbidity | Historical ICD or CB (%) | 32 (28.32) | 10 (37.03) | 0.53 |
|  | Premorbid impulsive personality (%) | 8 (7.08) | 6 (22.2) | **0.04** |
|  | Familiar history of ICD or BD (%) | 19 (16.8) | 1 (3.7) | 0.15 |
|  | Family history of PD (%) | 31 (27.43) | 11 (40.74) | 0.26 |
| Comorbidity | Antidepressant treatment (%) | 20 (18.3) | 7 (24.1) | 0.53* |
|  | Anxiolytic treatment  N (%) | 18 (15.9) | 5 (18.5) | 0.68* |
|  | Antipsychotic treatment  N (%) | 4 (3.5) | 0 (0) | 0.99* |
|  | Smoke  N (%) | 42 (37.7) | 7 (25.9) | 0.38 |
|  | Alcohol intake  N (%) | 26 (23) | 1 (3.7) | **0.04** |
| Results for univariate tests: chi-square test, t-test or Wilcoxon test.  *Results for multivariate analysis: multivariate linear regression or multivariate logistic regression adjusted by age, years of disease duration.  ^1^Cultre level (Elementary / High School / Universitary), ^2^Life style (Married/Married with children/Others/Alone), ^3^Habitat (Rural/Semiurban/Urban)  ICDs: impulse control disorders, CBs: compulsive behaviour, N: number of subjects, M: male, F: female, PD: Parkinson’s disease | | | | |

Table 5. Motor and non motor evaluations in Parkinson’s disease according to the presence of impulse control disorders in patients with age at onset under 50 years old.

|  |  | **ICDs negative** | **ICDs positive** | **P value** |
| --- | --- | --- | --- | --- |
| Number of subjects |  | 113 | 27 | - |
| Family history PD | N | 31 (27.4) | 11 (40.74) | 0.26 |
| Age at onset | Age (Mean ± SD) | 44.37±5.5 | 43.44±5.98 | 0.47 |
| Disease evolution time | Years (Mean ± SD) | 7.33±5.9 | 6.66±4.37 | 0.99 |
| UPDRS  Score (Mean ± SD) | II OFF | 2.9±2.12 | 3.61±2.21 | 0.06* |
|  | II ON | 1.68±1.94 | 1.2±1.31 | 0.47* |
|  | III OFF | 24.8±15.52 | 26.42±13.33 | 0.35* |
|  | III ON | 15.94±8.67 | 16±5.79 | 0.98* |
|  | IV | 2.8±2.98 | 2.52±2.22 | 0.66* |
| Phenotype (undetermined/PIGD/Tremor) | N | 18/51/43 | 5/12/10 | 0.95 |
| Dominant hemibody | N | 6/32/17/32/25 | 0/7/8/6/6 | 0.36 |
| Motor fluctuations | N (%) | 51 (45.1) | 9 (33.3) | 0.25* |
| Dyskinesias | N (%) | 26 (23.21) | 4 (14.8) | 0.33* |
| NMSS | Score (Mean ± SD) | 44.72±40.29 | 65.89±50.2 | **0.01*** |
| Pain | N (%) | 71 (62.8) | 18 (66.6) | 0.71* |
| PDSS | Score (Mean ± SD) | 107.41±32.56 | 101.55±30.85 | 0.34* |
| REM sleep behaviour disorder | N (%) | 41 (36.28) | 13 (48.15) | 0.16* |
| BECK (depression yes) | N (%) | 54 (47.7) | 15 (55.5) | 0.39* |
| BECK- Depression Inventory | Score (Mean ± SD) | 9.5±8.55 | 13.8±9.8 | **0.02*** |
|  | Subclinic/minor/major | 21/10/23 | 6/4/5 | 0.6 |
|  | No depression vs subclinic | 59/21 | 12/6 | 0.47* |
|  | No depression vs minor | 59/10 | 12/4 | 0.32* |
|  | No depression vs major | 59/23 | 12/5 | 0.79* |
| FOG | Score (Mean ± SD) | 4.24±4.71 | 7.07±6.25 | **0.002*** |
| MMSE | Score (Mean ± SD) | 29.24±1.01 | 29.4±0.88 | 0.59* |
| PD-CRS | Score (Mean ± SD) | 98.088±15.76 | 101.59±16.79 | 0.46* |
|  | Non CI/MCI/dementia | 95/17/1 | 24/3/0 | 0.77 |
|  | Fronto-subcortical | 69.83±14.93 | 73.4±15.75 | 0.38* |
|  | Posterior cortical | 28.25±2.89 | 28.18±2.62 | 0.67* |
| NPI (severity x frequency) | Score (Mean ± SD) | 7.36±10.26 | 8.87±10.62 | 0.51* |
| PDQ-39 | Score (Mean ± SD) | 19.69±1.95 | 25.35±16.03 | **0.05*** |
| WHOQOL-8 | Score (Mean ± SD) | 7.07±1.65 | 6.63±1.76 | 0.22* |
| Results for univariate tests: chi-square test, t-test or Wilcoxon test.  *Results for multivariate analysis: multivariate linear regression or multivariate logistic regression adjusted by age, years of disease duration.  ICDs: impulse control disorders, FOG: Freezing of Gait Scale, UPDRS: Unified Parkinson’s Disease Rating Scale, N: number of subjects, NMSS: Non Motor Symptoms Scale, NPI: Neuropsychiatry Inventory, PD: Parkinson’s disease, PD-CRS: Parkinson’s Disease Cognitive Rating Scale, PDQ-39: 39 items Parkinson’s Disease Questionnaire, PDSS: Parkinson’s Disease Sleep Scale, REM: Rapid Eye Movements, SD: standard deviation, WHOQOL-8: 8 items World Health Organization Quality of Life. | | | | |

Table 6. Principal investigators and sites included in COPPADIS Study Group.

| **Principal Investigator; PI’s participating site** | | **City** |
| --- | --- | --- |
| 1. | Diego Santos García; Neurology Section, Hospital Arquitecto Marcide, Complejo Hospitalario Universitario de Ferrol (CHUF). | Ferrol (A Coruña) |
| 2. | (1) Oriol de Fábregues-Boixar Nebot and (2) Jorge Hernández Vara; Movement Disorders Unit, Neurology Service, Hospital Universitario Vall d’Hebron. | Barcelona |
| 3. | Carmen Borrue Fernández; Movement Disorders Unit, Neurology Service, Hospital Infanta Sofía. | Madrid |
| 4. | Pablo Mir Rivera; Movement Disorders Unit, Neurology and Clinical Neurophysiology Service, Instituto de Biomedicina de Sevilla, Hospital Universitario Virgen del Rocío, CSIC and Universidad de Sevilla. | Seville |
| 5. | Maria José Martí Domenech; Parkinson’s and Movement Disorders Unit, Neurology Service, Instituto Clínico de Neurociencias, Hospital Clínic. | Barcelona |
| 6. | Miquel Aguilar Barberá; Movement Disorders Unit, Hospital Universitario Mutua de Terrassa. | Barcelona |
| 7. | Beatriz Tijero Merino; Functional Neurology and Parkinson’s Disease Unit, Hospital de Cruces. | Bilbao |
| 8. | José Chacón Peña; Neurology Unit, Hospital Infanta Luisa. | Seville |
| 9. | (1) Manuel Seijo Martínez and (2) Iria Cabo López; Neurology Section, Hospital de Pontevedra. | Pontevedra |
| 10. | Víctor Puente Périz; Movement Disorders Unit, Neurology Service, Hospital del Mar. | Barcelona |
| 11. | Inés Legarda Ramiréz; Neurology Service, Hospital Universitario Son Espases. | Palma de Mallorca |
| 12. | Francisco Carrillo Padilla; Neurology Service, Hospital Universitario de Canarias, San Cristóbal de la Laguna. | Santa Cruz de Tenerife |
| 13. | Lydia López Manzanares; Movement Disorders Unit, Neurology Service, Hospital La Princesa. | Madrid |
| 14. | Caridad Valero Merino; Neurology Unit, Hospital Arnau de Vilanova. | Valencia |
| 15. | Jaime Kulisevsky Bojarski; Movement Disorders Unit, Neurology Service, Hospital de Sant Pau. | Barcelona |
| 16. | José Manuel García Moreno; Movement Disorders Unit, Hospital Universitario Virgen Macarena. | Seville |
| 17. | Benito Galeano Bilbao; Neurology Section, Hospital Universitario de Ceuta. | Ceuta |
| 18. | Nuria Caballol Pons; Movement Disorders Unit, Consorci Sanitari Integral, Hospital Moisés Broggi. | Sant Joan Despí |
| 19. | Mari Cruz Rodríguez Oroz; Hospital Universitario Donostia, Instituto de Investigación Biodonostia. | San Sebastián |
| 20. | María Iciar Gastón Zubimendi; Movement Disorders Unit, Neurology Service, Complejo Hospitalario de Navarra. | Pamplona |
| 21. | Pilar Sánchez Alonso; Neurology Service, Hospital Puerta de Hierro. | Madrid |
| 22. | Esther Cubo Delgado; Neurology Service, Complejo Asistencial Universitario de Burgos. | Burgos |
| 23. | Lydia Vela Desojo; Neurology Unit, Fundación Hospital de Alcorcón. | Alcorcón (Madrid) |
| 24. | Maria José Catalán Alonso; Movement Disorders Unit, Neurology Service, Hospital Clínico San Carlos. | Madrid |
| 25. | Luis Manuel López Díaz; Neurology Section, Hospital de Burela. | Burela (Lugo) |
| 26. | Maria Gema Alonso Losada; Neurology Service, Hospital Meixoeiro, Complejo Hospitalario Universitario de Vigo (CHUVI). | Vigo (Pontevedra) |
| 27. | Nuria López Ariztegui; Movement Disorders Unit, Complejo Hospitalario de Toledo. | Toledo |
| 28. | Mónica Kurtis Urra; Movement Disorders Unit, Neurology Service, Hospital Ruber Internacional. | Madrid |
| 29. | Jon Infante Ceberio; Movement Disorders Unit, Neurology Service, Hospital Universitario Marqués de Valdecilla. | Santander |
| 30. | Sonia Escalante Arroyo; Neurology Service, Hospital de Tortosa Verge de la Cinta (HTVC). | Tortosa (Tarragona) |
| 31. | Juan Carlos Martínez Castrillo; Movement Disorders Unit, Neurology Service, Hospital Ramón y Cajal. | Madrid |
| 32. | José Matías Arbelo González; Movement Disorders and Parkinson’s Disease Unit, Neurology Service, Hospital Universitario Insular de Gran Canaria. | Las Palmas de Gran Canaria |
| 33. | René Ribacoba Montero; Movement Disorders Unit, Neurology Service, Hospital Central de Asturias. | Oviedo |
| 34. | Jessica González Ardura; Neurology Service, Hospital Universitario Lucus Augusti (HULA). | Lugo |
| 35. | Javier López del Val; Movement Disorders Unit, Neurology Service, Hospital Clínico Universitario Lozano Blesa. | Zaragoza |
| 36. | María Asunción Ávila Rivera; Movement Disorders Unit, Consorci Sanitari Integral, Hospital General de L’Hospitalet. | L’Hospitalet de Llobregat |
| 37. | Hortensia Alonso Navarro; Neurology Section, Hospital Universitario del Sureste, Madrid. | Madrid |
| 38. | Berta Solano Vila; Neurology Service, Hospital Josep Trueta and Parc Martí i Juliá, Girona. | Girona |
| 39. | Juan García Caldentey; Neurology Unit, Hospital Quirón Palmaplanas. | Palma de Mallorca |
| 40. | Ana Rojo Sebastián; Parkinson’s and Abnormal Movement Unit, Neurology Service, Hospital Universitario Príncipe de Asturias. | Alcalá de Henares (Madrid) |
| 41. | Silvia Martí Martínez; Neurology Service, Hospital General de Alicante. | Alicante |
| 42. | José Andrés Domínguez Morán; Neurology Unit, Hospital de la Rivera. | Alcira (Valencia) |
| 43. | Irene Martínez Torres; Movement Disorders Unit, Neurology Service, Hospital La Fe. | Valencia |
| 44. | María Álvarez Sauco; Neurology Service, Hospital General Universitario de Elche. | Elche (Alicante) |
| 45. | Cristina Prieto Jurczynska; Movement Disorders Unit, Hospital Infanta Elena-Hospital Rey Juan Carlos-Hospital Collado Villalba, Madrid. | Madrid |

The local Ethics Committee at each center approved the participation in the study.
